# Trends in Antimicrobial Consumption in Tertiary Care Hospitals in Costa Rica from 2017 to 2021: A Comparative Analysis of Defined Daily Doses per 100 Bed Days and per 100 Discharges

**DOI:** 10.3390/antibiotics13100939

**Published:** 2024-10-06

**Authors:** Cristina Fernández-Barrantes, Allan Ramos-Esquivel, Luis Esteban Hernández-Soto, Manuel Ramírez-Cardoce, Luis David Garro-Zamora, Jose Castro Cordero, Santiago Grau

**Affiliations:** 1 Department of Pharmacology, Faculty of Medicine, Universitat Autònoma de Barcelona, 08193 Barcelona, Spain; cristina.fernandezbarr@autonoma.cat; 2 Department of Pharmacy, Hospital San Juan de Dios, Caja Costarricense de Seguro Social, San José 10103, Costa Rica; 3 School of Medicine, University of Costa Rica, San José 11501, Costa Rica; allan.ramos@ucr.ac.cr; 4 Faculty of Pharmacy, University of Costa Rica, San José 11501, Costa Rica; luis.hernandez@ucr.ac.cr; 5 Infectious Diseases Unit, Hospital San Juan de Dios, Caja Costarricense de Seguro Social, San José 10103, Costa Rica; meramirec@ccss.sa.cr; 6 Department of Pharmacy, Hopital México, Caja Costarricense de Seguro Social, San José 10107, Costa Rica; ldgarro@ccss.sa.cr; 7 Infectious Diseases Unit, Hospital México, Caja Costarricense de Seguro Social, San José 10107, Costa Rica; jancastro@ccss.sa.cr; 8 Department of Pharmacy, Hospital del Mar, Parc de Salut Mar, 08003 Barcelona, Spain

**Keywords:** antimicrobial consumption, antimicrobial stewardship, defined daily dose

## Abstract

**Background**: Antimicrobial consumption (AMC) data in Latin America are scarce and usually spread out across different sources used to make AMC calculations, making it difficult to both standardize and compare regions through similar time frames. The main objective was to analyze AMC trends in Social Security tertiary care hospitals in Costa Rica in the period spanning January 2017 to December 2021, using both the defined daily dose (DDD)/100 bed days and DDD/100 discharges. **Methods**: This is a retrospective observational study of antimicrobial consumption. Global consumption trends were calculated and expressed as DDD/100 bed days and DDD/100 discharges. Trends in antimicrobial consumption were analyzed using a simple linear regression model to determine potential differences in antimicrobial usage throughout the study’s duration. **Results**: A statistically significant increase in the consumption expressed in DDD/100 discharges was observed in the following groups: carbapenems, 7.6% (trend: 64.68, *p* < 0.0001), trimethoprim-sulfamethoxazole: 12.6% (trend: 16.45, *p* < 0.0001), quinolones 9.4% (trend: 36.80, *p* = 0.02), vancomycin 2.0% (trend: 16.30, *p* = 0.03), echinocandins: 6.0% (trend: 15.17, *p* = 0.01) and azole antifungals: 12.10% (trend: 102.05, *p* < 0.0001). Additionally, a statistically significant increase of 10.30% in the consumption of azole antifungals expressed in DDD/100 bed days was observed (*p* = 0.0008). In contrast, a statistically significant decrease in consumption, expressed in DDD/100 discharges, was identified for cephalosporins −6.0% (*p* < 0.0001) and macrolides −16.5% (*p* < 0.0001). Macrolides also showed a downward trend in consumption, as expressed in DDD/100 bed days (−14.3%, *p* < 0.0001). According to World Health Organization (WHO) access, watch and reserve (AWaRe) classification trend analysis, only the reserve group showed a statistically significant upward change of 9.2% (*p* = 0.016). **Conclusions**: This five-year analysis demonstrated trends over time in overall antimicrobial consumption measured in DDD/100 bed days and DDD/100 discharge rates that correlate. In general, for all antimicrobials, after the implementation of antimicrobial stewardship programs (ASP), a downward trend is reported; in contrast, during the COVID-19 pandemic the AMC shows a general upward trend. The comparison between DDD/100 bed days and DDD/100 discharges allows for complementary comparisons to be made regarding antimicrobial exposure in a clinical setting.

## 1. Introduction

Antimicrobial resistance (AMR) poses a major threat to global human health [1]. A study published in 2019 established a link between AMR and approximately 4.95 million deaths, with increased projections to 10 million deaths per annuum by 2050 [2]. Liberal antimicrobial use is one of the main drivers of AMR; hence, the surveillance and optimal use of the latter are among the key strategies use to mitigate AMR and are included in the five main objectives of the World Health Organization (WHO)’s Global Action Plan (GAP) for AMR [3].

Antimicrobial usage primarily takes place in outpatient and community care. Nevertheless, in hospital environments, the intensity of antimicrobial consumption is significantly greater as vulnerable patients situated closely together, which increases the likelihood of the emergence and spread of resistant pathogens [4].

Additionally, fungal diseases represent a growing concern, with a significant impact on morbidity and mortality, as has been previously observed with bacteria; it is known that the improper use of antifungals poses a selective pressure that is a key driver for the emergence and dissemination of antifungal-resistant strains in clinical settings [5,6].

Monitoring antimicrobial consumption (AMC) in health facilities is an important element of all antimicrobial stewardship programs (ASP) [7]. In facilitating the standardizing of results and the ability to compare consumption information across time and different hospitals, the most widely implemented system is the defined daily dose (DDD), according to the WHO Anatomical Therapeutic Chemical (ATC) system [8,9,10]. Standardizing measures of hospital activity and hospital consumption data must correspond strictly to the time people are under surveillance and the hospital activity units covered (bed days and/or discharges) [4,9]. The purpose is to identify areas in need of improvement and assess interventions by comparing hospitals of the same complexity and by evaluating time series data from a single center, while providing support for studying ecological effects [8,11].

Another antimicrobial stewardship (AMS) tool used to monitor AMC is the WHO access, watch and reserve (AWaRe) classification of antibiotics, developed in 2017. This tool classifies antibiotics into three groups, taking into account the impact of different antibiotics on AMR, to emphasize the importance of their appropriate use [4]. The access group includes first or second choices for the treatment of bacterial infections while minimizing the potential of AMR. Antibiotics in the watch group are recommended as a first or second choice for the empiric treatment of a specific, limited number of infective syndromes, but have, in general, a strong potential for generating AMR in bacteria [11]. The reserve group includes antibiotics and antibiotic classes that should be reserved for the treatment of confirmed or suspected infections due to multidrug-resistant (MDR) organisms; in many scenarios these agents are the only therapeutic option [4].

In Costa Rica, a country with a total population of 5,044,197 inhabitants, public healthcare facilities belong to the Social Security, Caja Costarricense de Seguro Social (CCSS), network model, which is independent from the Ministry of Health. This model is based on three attention levels, where the third level stands as the one of maximum complexity. Furthermore, an institutional medicines policy has been developed based on the WHO List of Essential Medicines model, which seeks to satisfy most of the population’s health necessities. Medication is accessible to patients through the universal coverage provided by CCSS [12,13,14,15].

Nonetheless, the Ministry of Health of Costa Rica established its own National Action Plan to Mitigate Antimicrobial Resistance in 2018, under which the surveillance of AMC in healthcare settings is mandatory. This initiative is yet to be implemented, thus rendering knowledge on this topic scarce across Costa Rica and its neighboring Central American nations [16,17].

Antimicrobial stewardship programs have been implemented in different healthcare hospitals in Costa Rica during the past decade; one example is Clinica Biblica Hospital, a private institution with 68 beds in San José, Costa Rica, where, in 2015, an AMS was implemented. The program has been continuously monitored by a multidisciplinary team, consisting of a clinical pharmacist (director), an infectious disease expert, a microbiologist and the hospital’s assistant chief medical officer. In addition, since 2018, AMSs have been implemented in Social Security tertiary care hospitals following a multidisciplinary approach; the main objectives have focused on educational strategies, the application and tracking of antimicrobial guidelines, diagnostic stewardship, monitoring AMC and evaluating economic outcomes [18,19,20].

The objective of the present study was to describe and analyze antimicrobial consumption trends in Social Security’s tertiary care hospitals across Costa Rica and throughout the period of 2017–2021, using both DDD/100 bed days and DDD/100 discharges as measurements of hospital activity.

## 2. Results

### 2.1. Indicators of Hospital Activity

Table 1 presents the mean of unadjusted DDDs, bed days, discharges and lengths of stay. During these five years, a slight increase in DDD was observed. In contrast, the number of bed days and discharges showed a downward trend (−0.13% and −0.15%, respectively). Contrastingly, the length of reported stays did not demonstrate a statistically significant change in the same time frame.

### 2.2. Antimicrobial Consumption Patterns and Trends in the Period 2017–2021

**Figure 1:**
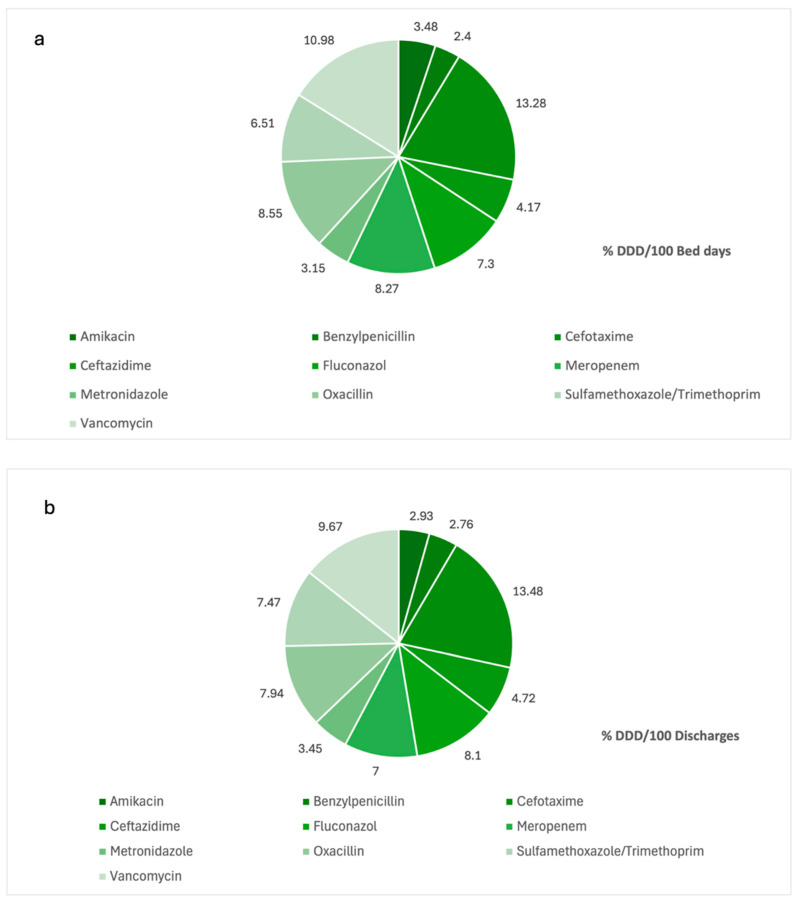
Consumption of antimicrobials ranked in top 10, expressed in % of DDD/bed days (**a**) and % DDD/100 discharges (**b**), from 2017 to 2021 in tertiary care hospitals in Costa Rica. DDD: defined daily dose.

Table 2 shows a description of the AMC patterns seen during each year of this study period; the top ten most common antimicrobials are reported in %DDD/100 bed days and %DDD/100 discharges. Figure 1 gives a graphical report of the top 10 most common antimicrobials used using both quantification indicators. According to our results, the top three most consumed antimicrobials are Cefotaxime, at 13% of the total of the AMC in both DDD/100 bed days and DDD/100 discharges; Vancomycin, at approximately 10% in both indicators; and Oxacillin, at 8.5% DDD/100 bed days and 8.1% DDD/100 discharges.

**Figure 2:**
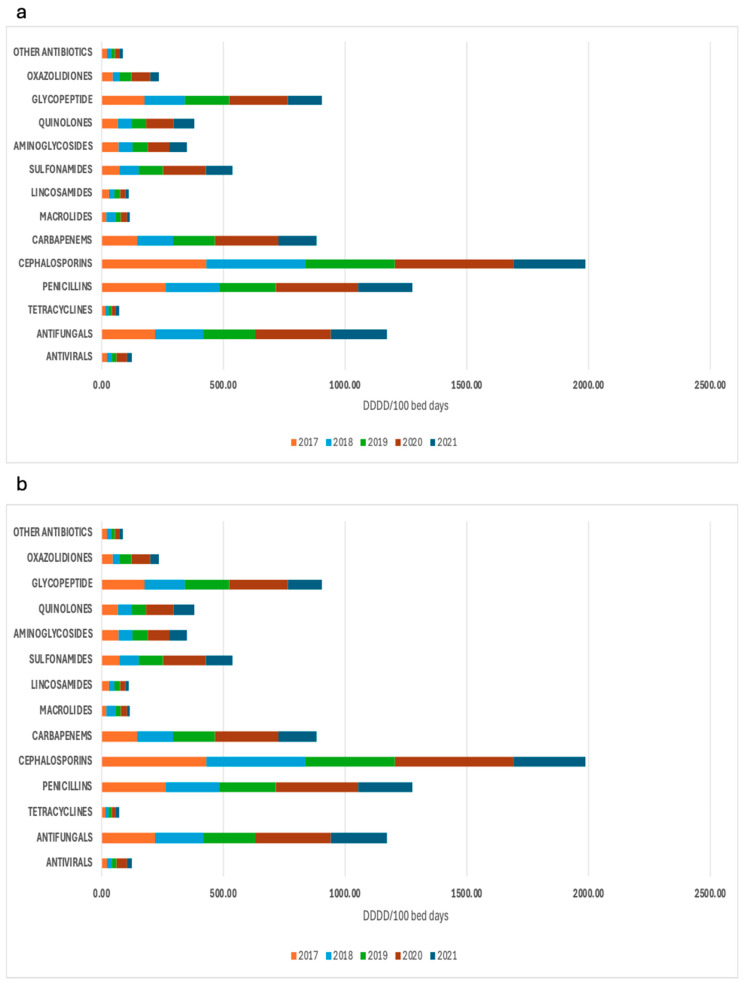
Total antimicrobial consumption per selected group, expressed in DDD/100 bed days (**a**) and DDD/100 discharges (**b**), from 2017 to 2021. DDD: defined daily dose.

As a complement to the patterns shown previously, a complete description of the AMC distribution per antimicrobial group is shown in Figure 2.

**Figure 3:**
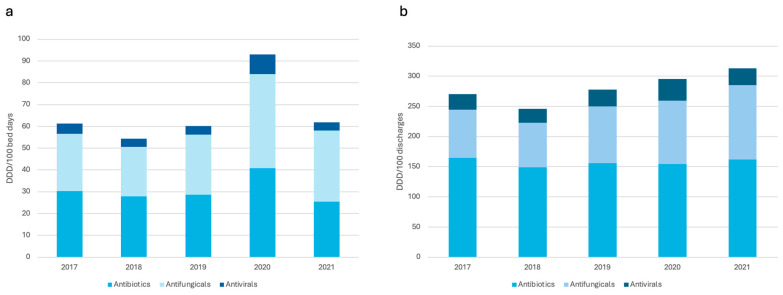
Total use of antimicrobials, expressed in DDD/100 bed days (**a**) and DDD/100 discharges (**b**), in tertiary care hospitals in Costa Rica during 2017–2021.

Global trends in antimicrobial consumption during this period are shown in Table 3. Evidently, the overall consumption of antimicrobial medication increased, in terms of DDD/100 discharges, from 270.63 to 313.25 (+2.97%, *p* = 0.0007). On the other hand, the consumption calculated in DDD/100 bed days showed no statistically significant changes, ranging from 61.35 to 61.78 (+0.14%, *p* = 0.31). In this general overview, despite antibiotics having a decreased use, they still showed a significantly increased use when compared to antivirals. However, no statistical difference was discerned across the period (Figure 3). Antifungals were the only group with a statistically significant increase in DDD/100 discharges: from 80.41 to 123.52 (+8.97%, *p* < 0.0001).

**Figure 4:**
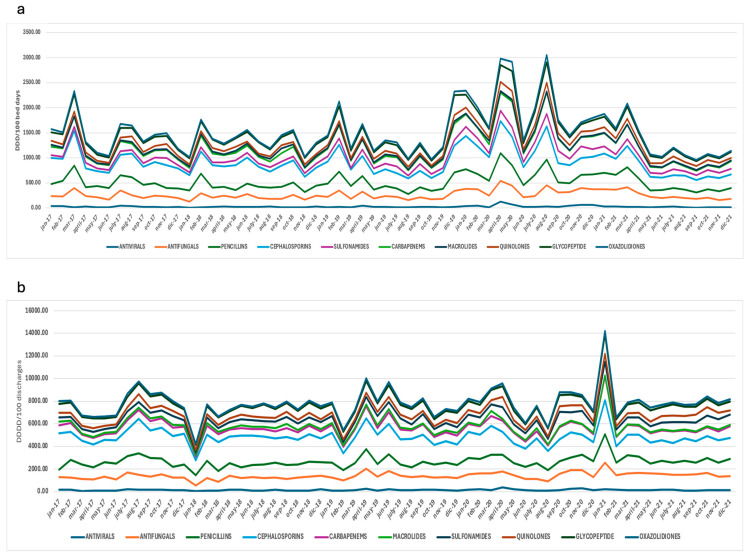
Monthly antimicrobial consumption trends for selected groups, in DDD/100 bed days (**a**) and DDD/100 discharges (**b**), from 2017 to 2021. DDD: defined daily dose.

Detailed AMC trends for the main antimicrobial groups are shown in Table 4. The following groups showed a statistically significant increase in consumption, measured in DDD/100 discharges: carbapenems, from 699.00 (2017) to 948.00 (2021), representing an increase of 7.6% (*p* < 0.0001); trimethoprim-sulfamethoxazole, from 475.60 (2017) to 772.85 (2021), with an increase of 12.6% (*p* < 0.0001); quinolones 9.4% (*p* = 0.02); vancomycin, from 814.88 (2017) to 878.52 (2021), with an increase of 2.0% (*p* = 0.03); echinocandins, from 156.94 (2017) to 189.48 (2021), increasing their consumption by 6.0%; and azole antifungals, from 647.18 (2017) to 1045.72 (2021), with an increase of 12.1% (*p* < 0.0001). Also, a statistically significant increase of 10.30% in the consumption of azole antifungals, expressed in DDD/100 bed days, was reported, from 116.52 (2017) to 163.64 (2021) (*p* = 0.0008). In contrast, a statistically significant decrease in consumption, expressed in DDD/100 discharges, was identified for cephalosporins—of −6.0%, from 2495.15 (2017) to 1946.72 (2021)—and macrolides—of −16.5%, from 360.60 (2017) to 174.26 (2021) (*p* < 0.0001). Macrolides also showed a downward trend in consumption, expressed in DDD/100 bed days, from 50.42 to 25.39 (−14.3%, *p* = 0.005). Figure 4 shows two stacked-line graphical descriptions of the monthly AMC trends for the most common antimicrobial groups used, expressed in DDD/100 bed days and DDD/100 discharges. The areas between the lines show the cumulative sum of all categories, presenting the total consumption of antimicrobials. After the implementation of ASP in our hospitals, a downward trend was reported, which is in contrast to during the first year of COVID-19 pandemic, where the AMC shows a general upward trend. According to our WHO AWaRe classification trend analysis, a statistically significant upward change of only 9.2% (*p* = 0.016) was shown. A complete description of the AWaRe classification trends is reported in Table 5.

## 3. Discussion

Latin American countries do not regularly measure their antimicrobial consumption, and only a few occasionally review the overall consumption of antibiotics within their territory [17]. Due to these inconsistencies, data in this region are scarce and usually spread out over different sources used to make AMC calculations, making it difficult to both standardize and compare regions through similar time frames.

This is the first multicentric study with a standardized methodology that analyzes antimicrobial consumption trends in Social Security acute tertiary care hospitals in Costa Rica. Furthermore, another advantage this study presents is that it was carried out with data from the two hospitals in Costa Rica with the greatest complexity. Monitoring the consumption of antimicrobials in the hospital setting is a necessary measure, both for cost optimization reasons and to develop further strategies to suppress the appearance of resistant strains. Additionally, this study may become useful when evaluation stewardship programs follow results and trends that have been presented previously [9,21].

Variations in in-hospital antimicrobial use may be due to several factors. Firstly, it may depend on both hospital and patient characteristics, in addition to any pre-existing antibiotic policies, as well as the physician’s education or the beliefs held by different healthcare systems. However, a substantial part of the differences in AMC metrics may be the result of differences in the methods used to measure antimicrobial use [22].

The DDD is the most accepted measure of AMC, and it refers to the average maintenance dose assumed for a drug´s main indication in adults [20]. The ATC/DDD system from the WHO allows for data collection at an aggregated level and avoids the need for individual-level data. This versality allows resource-limited countries to leverage existing data sources to develop sustainable AMC monitoring systems [17].

Even though the ATC/DDD system for all drugs has been available since the 1980s, it was not widely used, and in some cases it was misunderstood. This resulted in widespread confusion, due to misinformed publications, along with the incomplete and unspecified publication of antibiotic guidelines [22,23].

Most publications that quantify antimicrobial consumption trends in the hospital setting using this methodology use DDD/100 bed days as the standard denominator. This parameter reflects the hospital’s exposure to antimicrobials, but does not describe either number or proportion of patients treated with antimicrobials, while parameters such as DDD/100 discharges provides information on the exposure per patient and helps to interpret consumption trends over time, as it shows changes in hospital activity [21,24].

The most common antimicrobials consumed (Figure 1 and Table 2) in our tertiary care hospitals from 2017 to 2021 were cefotaxime, vancomycin, oxacillin and fluconazole. The high proportion of cefotaxime and third-generation cephalosporins in general may be explained by different reasons; for example, due to its broad-spectrum activity, cefotaxime is prescribed as an empirical treatment option, and also it is believed that cephalosporins tend to be less allergenic compared to penicillins [25].

Recently, prescription numbers for cephalosporins from a national healthcare database from the United States reported that 47.5% of all antibiotics used corresponded this antimicrobial family, exceeding the number of narrow- and wide-spectrum penicillins (39.7% of total antibiotics) recorded by this national healthcare database from 2004 to 2014 [26].

Although these results cannot be directly compared due to the discrepancy between the methods employed to quantify their consumption, they do in some way provide a reference for how frequent the consumption of cefotaxime and third-generation cephalosporins are in general.

In the same way, the infections caused by methicillin-resistant *Staphylococcus aureus* (MRSA) and methicillin-sensible *S. aureus* (MSSA) are frequent in nosocomial environments and a global threat to AMR. Vancomycin remains one of the first-line antibiotics for the treatment of MRSA and oxacillin is the same for MSSA. However, resistance to these agents has emerged in recent years due to the misuse of these antibiotics, especially in empirical treatment scenarios [25].

This study includes the COVID-19 pandemic time frame, 2020–2021; one of the main consequences of this pandemic was the imbalance associated with antimicrobial use, probably due to our initially poor knowledge of the burden of the disease. One important finding is the rise in the incidence of invasive aspergillosis and candidemia during the pandemic; this observation could account for the rise in fluconazole consumption seen in our hospitals, as well as in acute care hospitals in Catalonia [27].

The results from this study showed that trends in overall antimicrobial consumption and consumption per antimicrobial group in DDD per 100 bed days consistently correlate with trends in DDD per 100 discharges over time (Table 3 and Table 4). Differences in trends between the two units of measurement seem to be the result of changes in resource indicators over time. Both bed days and discharges underwent statistically significant decreases during the study period; however, the length of stay remained stable.

Comparing the AMC results with other hospitals in different geographical regions remains a challenge, given the heterogeneity in the methodologies employed in this type of study. Despite this challenge, some similarities were found in the literature.

The WHO Global Antimicrobial Resistance and Use Surveillance System (GLASS) report from 2022 provided information from 36 countries, territories and areas (CTAs) on the status of AMC surveillance implementation, with data available from 2020 [28,29]. However, interpreting the data from this report becomes a challenge, given the small number of reporting CTAs and the wide variety of AMC data collection methods. Data from 26 CTAs included the analysis of AMC, covering all antimicrobials. The findings showed that antibacterials were the most often consumed. Although antibacterial use varied across CTAs, penicillin consumption was 34% of total antibacterial use, with its the highest rate a median of 7.1 (range, 1.0–24.2 DDD) per 1000 inhabitants per day. Third-generation cephalosporins (J01DD) were the most prescribed subgroup within cephalosporins in 12 CTAs, with a median relative consumption of 43% (range, 9–99%). Systemic antifungals (J02, D01B) and antivirals (ATC J05) were reported by 12 CTAs, with median values of 0.9 (range 0.03–3.35) and 1.7 (range, 0.8–3.36) DDD per 1000 inhabitants per day, respectively [28,29].

According to the data from the 2022 Annual Epidemiological Report by the European Centre for Disease Prevention and Control, the reported penicillins had the highest antibiotic use in eighteen Europeans countries, while cephalosporins and other beta-lactams had the highest use in nine countries [30]. From 2013 to 2022, there were statistically significant increases in the hospital AMC of tetracyclines, sulfonamides and trimethoprim, as well as other antibacterials. However, penicillin, cephalosporins and other beta-lactams, macrolides, lincosamides and streptogramins had no significant trend changes.

In addition to the WHO’s world report and the European data, a study was conducted in 2019 across Latin America. This study showed highly variable rates of AMC, from 1.91 DDD/1000 inhabitants in private institutions in Paraguay to 36.26 DDD/1000 inhabitants in Argentina. Furthermore, this study demonstrated that penicillin was the most consumed group in all countries included, except Paraguay, while macrolides and lincosamides were ranked second across the board [17].

In our analysis, we included information from specific regions and countries. For example, in Catalonia, a region of Spain with 7.5 million inhabitants, the most frequently used groups of antibiotics in the period from 2007 to 2019 were penicillin (J01C), quinolones (J01M), cephalosporins (J01DB, J01DC, J01DD and J01DE), other antibiotics (J01X) and carbapenems (J01DH). In 2009, these antibiotic groups represented 88.9% of the total antibiotics consumed in this region. Additionally, a study conducted in México in secondary- and tertiary-care-level hospitals during 2016 and 2017, where AMC was quantified using DDD/100 occupied bed days, showed that the antimicrobials with higher consumption rates were cephalosporines, carbapenems and vancomycin [31].

A study conducted between 2012 and 2020 in Gansu, China, found the three antibiotics most often consumed during this period; first, accounting for 45.15% of AMC, was the penicillin group (J01C), followed by macrolides, lincosamides and streptogramins (J01D), accounting for 31.40%, and then cephalosporins (J01D) at 11.99% [32]. Similarly, our study found that the antibiotics most often used were cephalosporins, penicillins like oxacillin and carbapenems like meropenem.

The Canadian Antimicrobial Resistance Surveillance System Report published in 2022 tracked the country’s AMC trend from 2017 to 2021. The report showed that, at the national level, AMC dropped by 26.9%, from 17.0 to 12.5 DDD per 1.00 inhabitants per day. The largest drop was seen at the onset of the COVID-19 pandemic (2020 to 2021). Declines were noted in both the community 27.0% (15.7 to 11.4 DDDs per 1000 inhabitants per day) and the hospital sector 25.3% (1.4 to 1.0 DDDs per 1000 inhabitants per day) [33].

A study from Colombia conducted from 2019 to 2020 demonstrated changes in the reported of DDD/100 occupied beds for specific antibiotics, such as ceftriaxone in ICU wards (2019: 17,584; 2020: 19,857). In addition, the antibiotic consumption reported increased for piperacillin/tazobactam (from 91,606 to 94,076), ertapenem (from 5793 to 6051) and cefepime (26,809 to 38,780) from 2019 to 2020 [34]. Our results are in line with these findings. An increased consumption of carbapenems and penicillin was observed. Despite this, our study is not fully comparable, given a different methodology was employed.

One of the most significant findings is an increase of 7.6% in the Carbapenem use trend, expressed in DDD/100 discharges. This overall increase in the use of carbapenems could lead to the appearance of carbapenem-resistant bacteria. COVID-19 could be partially culpable for this increase, although this is yet to be confirmed. During the early stages of the COVID-19 pandemic, most hospitalized patients with reported, suspected or confirmed secondary bacterial infections were treated with broad-spectrum antibiotics on many occasions, regardless of the severity of the infection [35].

Additionally, a study from Catalonia in reported a worrisome increase in the use of Carbapenem by 88.43%, from 3.37 DDD/100 Patient Days (PDs) to 6.35 DDD/100 PD (*p* < 0.001). This eight-year surveillance study (2008 to 2015) showed a sustained and widespread increase in carbapenem use. This trend is consistent with tendencies observed in other European countries. Meropenem was the most prescribed carbapenem, while the consumption of imipenem-cilastatin showed a downward trend [36].

A peculiar finding was the downward trend in cephalosporins’ consumption. A possible explanation is the substitution of cephalosporins with broad-spectrum antibiotics during the pandemic period. Since 2018, antimicrobial stewardship programs have been in operation in the hospitals included in this study, promoting de-escalation and shortened therapies. Furthermore, a downward trend in macrolide consumption was observed. Also, a downward trend in macrolide consumption was seen with both AAPC indicators: −14.3% DDD/100 bed days and −16.5% DDD/100 discharges. This downward trend may be attributed to the fact that the increase in macrolide resistance minimizes its potential utility in the management of *Streptococcus pneumoniae* infections. Studies suggest that macrolide resistance in *S. pneumoniae* varies widely across different regions, with global resistance rates ranging between 30% and 50% [37].

In contrast, an upward trend was seen with sulfamethoxazole and trimethoprim due to an internal policy on empirical prescriptions established by the AMS program in 2018, which restricted the use of some antibiotics with a high prevalence of resistance such as cephalexin, cephalothin, clindamycin, gentamicin and oxacillin; the prescription of these antibiotics must be justified with an antibiogram or according to pre-existing protocols.

Also, a 9.4% increase in quinolones’ DDD/100 discharges has been reported. Quinolones are often used because of their high bioavailability and broad-spectrum antimicrobial activity. In contrast, a study of the trends and patterns of antimicrobial consumption in Japan from 2004 to 2016 indicates that the consumption of oral quinolones has stabilized since 2012, probably due to an increase in fluroquinolone resistance in *Escherichia coli*, which might have restricted its use for urinary tract infections in recent years [25].

Our antimicrobial consumption trend analysis also reported a 2% average increase in DDD/100 discharges for vancomycin; as explained above, this antibiotic is widely used as first-line option for MRSA and *Clostridiodes difficile* infections, which are important healthcare-associated infections in our hospitals [38].

**Figure 5:**
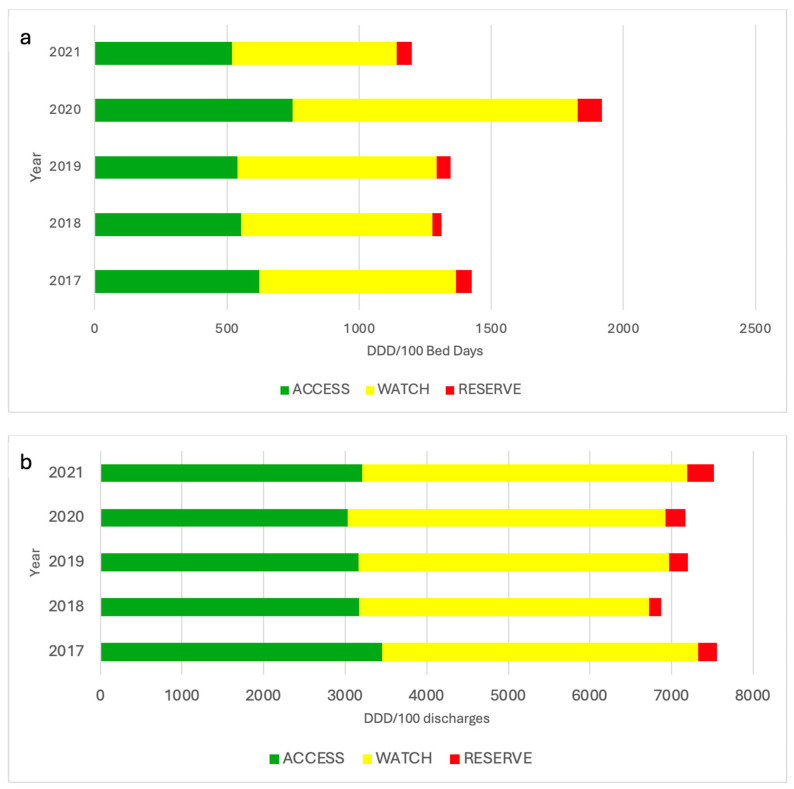
Antibiotic consumption according to WHO AWaRe classification, expressed in DDD/100 bed days (**a**) and DDD/100 discharges (**b**), from 2017 to 2021. DDD: defined daily dose; WHO-AWaRe: World Health Organization antibiotic classification (access, watch and reserve).

An additional analysis of antimicrobial consumption trends using the AWaRe classification was performed. Firstly, the watch group is the most prominently consumed; this is because agents like cefotaxime, ceftazidime, quinolones and carbapenems are included in this category (Figure 5). On the other hand, there was found to be an average increase of 9.2% (*p* = 0.016) in consumption mostly in the reserve group. An increase in the reserve group is worrying because these antibiotics may be related to the emergence of AMR. In hospitals this could indicate inadequate prescribing; however, it is important to note that these agents were frequently used during the COVID-19 pandemic, which could account for their upward trend. Close monitoring for timely stewardship interventions is needed for the watch and reserve groups [38].

Moreover, an increasing trend in the consumption of azole antifungals was reported. This finding is highly concerning, especially considering the implications of antifungals concerning the morbidity and mortality of mycoses. The clinical consequences of this resistance are observed in treatment failures and changes in the prevalence of fungal species like *Candida auris*, which is spreading at an alarming rate throughout United States healthcare facilities and is considered an “urgent antimicrobial resistance threat” by its health agency [39].

It is important to mention that this study is not without limitations. Firstly, we cannot be certain that the prescribing practices observed were representative of other hospitals of lower complexity in Costa Rica. Additionally, the results could have been influenced by the COVID-19 pandemic, like other studies. Furthermore, other analyses of hospitals of different complexity, other reference hospitals and private institutions and stratifying services into ICU, medical and surgical services would improve the quantity of the data obtained and allow more robust conclusions to be formulated. Moreover, in future studies, other statistic tests could be used to analyze antimicrobial consumption trends, such as the Kendall–Mann. One of the most reported methodologies in the literature used to monitor AMC at a hospital level is the Point Prevalence Survey of hospital antibiotic use. Even though these surveys are also useful for identifying AMS priorities, they do not allow for the evaluation of the duration of therapy, and more research is needed to understand current antibiotic prescribing patterns with respect to their duration, because this has been a major driver of inappropriate antibiotic use [40].

## 4. Materials and Methods

### 4.1. Setting and Study Design

This retrospective observational study was conducted in general tertiary care hospitals in the CCSS. According to the available data, two out of the three hospitals in this group were included: Hospital San Juan de Dios (635 beds) and Hospital México (466 beds). According to Social Security statistics, these hospitals provide care for approximately 85% of the Costa Rican population in terms of medical and surgical services, as well as providing intensive care units (ICUs) and ambulatory care facilities. This study was approved by the Costa Rican Social Security Ethics Committee: CEC-CENTRAL-CCSS (Protocol #R022-SABI-00319).

### 4.2. Data Collection

Data were collected monthly, starting from January 2017 and ending in December 2021, with the support of the pharmacy department software “Integrated Pharmacy System” (SIFA). Sixty-one antimicrobials for systemic use (J01, J02 and J05), according to the WHO-ATC classification, representing 46 different active principles, were included in the antimicrobial consumption quantification. Pediatric hospital wards, as well as units that do not generate high rates of discharge or occupied bed days (e.g., emergency services) were excluded from the data collection process. A description of the AMC per group and per specific antibiotic was included. Global consumption trends for antimicrobials groups and according to the WHO AWaRe classification were calculated and expressed in DDD/100 bed days and DDD/100 discharges, employing the WHO ATC-DDD Index 2023 for all five years as the assumed average maintenance dose per day for a drug used for its main indication adults. Before starting the study, responsible pharmacists were trained in WHO ATC-DDD calculation and the usage of the DDD Excel Calculator to guarantee a homogeneous collection of data [8,9].

### 4.3. Statistics Analysis

Trends in antimicrobial consumption were analyzed using a simple linear regression model to determine potential differences in antimicrobial usage throughout the period of the study. Simple linear regression was used to determine whether there was a significant difference between the calculated coefficient and zero.

The equation for the simple linear regression is

Y = β0 + β1X + ϵ

where Y: antimicrobial consumption rate in DDD/100 bed days or DDD/100 discharges (dependent variable), β0: the intercept (the estimated antimicrobial consumption when X = 0, or the starting point), β1: the slope (the estimated change in antimicrobial consumption per year), X: the year or time (independent variable), and ϵ: the error term (captures the variability not explained by the linear relationship)

If β1 (the slope) is positive, it suggests an increasing trend in antimicrobial consumption over the 5-year period; if β1 is negative, it indicates a decreasing trend; and if β1 is close to zero and not statistically significant, it suggests no significant trend in consumption over time.

The comparison of DDD, patient days, discharges and average bed days between 2017 and 2021 was carried out using the Wilcoxon signed-rank test.

Additionally, the average annual percent change (AAPC), with 95 percent confidence intervals, for different classes of antibiotics was incorporated into the analysis to measure the average percentage change in AMC over time, combining the year-to-year fluctuations into a single value.

*p* values of <0.05 were considered statistically significant. Statistical analysis was performed using Microsoft Excel (Microsoft Corporation, Redmond, WA, USA) and SAS Studio OnDemand for Academics (SAS Institute Inc., Cary, NC, USA).

## 5. Conclusions

Throughout this five-year study, the trends over time in overall antimicrobial consumption and consumption per group in terms of DDD per 100 bed days consistently correlate with the trends in DDD per 100 discharges. In general, for all antimicrobials, after the implementation of ASPs in our hospitals, a downward trend is reported. This is in contrast to the first year of the COVID-19 pandemic, where the AMC shows a general upward trend. According to the WHO AWaRe classification, the watch group accounts for the predominant proportion of consumption in our time series.

When analyzing specific antimicrobial groups, a concerning increase in carbapenem consumption of 7.6%, expressed in DDD/100 discharges, was observed. Moreover, other agents like sulfonamides and trimethoprim showed an increase of 12.6%, while quinolones showed an increase of 9.4% and vancomycin of 2.0%, a pattern possibly explained by the overuse of broad-spectrum antibiotics during the pandemic.

In contrast, a downward trend in the consumption of cephalosporins, −5.8% DDD/100 bed days and −6.0 DDD/100 discharges, and macrolides, −14.3% DDD/100 bed days and −16.5% DDD/100 discharges, was noticed. This decrease is possibly due to AMS interventions established in 2018 in the participating hospitals and due to the increased resistance of specific bacteria like *S. pneumoniae*, for which macrolides used to be one of the first options for treatment. The increasing use of azole antifungals (+10.3% DDD/100 bed days and +12.1% DDD/100 discharges) could lead to the development of fungal species resistant to the molecules available in our region. Furthermore, it is important to conclude that the comparison between DDD/100 bed days and DDD/100 discharges allows us to enhance our information about antimicrobial exposure in a clinical setting; with DDD/100 bed days it is possible to describe antibiotic exposure at a hospital level, but only DDD/100 discharges can indicate exposure at the patient level. Knowing the consumption of antimicrobials in a healthcare institution is the first step to improving their rational use and reducing bacterial resistance to these drugs. To determine this consumption, measurement standards must be standardized and validated in order to compare this rate within an institution and with other institutions.

## Figures and Tables

**Table 1:** Mean of defined daily doses (DDDs), bed days, discharges and lengths of stay in 2017 and 2021 among tertiary care hospitals in Costa Rica.

Mean	Year		Variation (%)	*p* Value
	2017	2021		
DDDs	**622.94**	890.74	0.43	**0.0004**
Bed days	14,478.46	12,555.54	−0.13	**0.0010**
Discharges	2011.75	1702.83	−0.15	**0.0004**
Length of stay	7.33	7.48	0.02	0.06

DDD: defined daily dose (unadjusted); %: difference between 2017 and 2021; statistically significant *p* values are in bold.

**Table 2:** Top 10 ranking of antimicrobial consumption, expressed in DDD/100 bed days and DDD/100 discharges.

DDD/100 Bed Days						DDD/100 Discharges					
Antimicrobials	2017	2018	2019	2020	2021	Antimicrobials	2017	2018	2019	2020	2021
Amikacin	53.68	41.5	52.41	77.95	61.6	Amikacin	239.96	183.35	231.96	223.16	363.19
Benzylpenicillin	54.12	42.16	34.97	38.9	27.93	Benzylpenicillin	322.02	264.01	222.38	182.34	176.69
Cefotaxime	**223.02**	**217.85**	**210.53**	**275.23**	**167.82**	Cefotaxime	**1215.25**	**1103.98**	**1152.61**	**1128.61**	**111.03**
Ceftazidime	80.99	64.99	62.13	108.61	70.3	Ceftazidime	435.95	322.11	354.17	414.51	473.03
Fluconazole	103.82	101.22	101.73	147.86	**147.18**	Fluconazole	563.25	**562.67**	**668.1**	696.02	**942.6**
Meropenem	130.24	**120.78**	**137.42**	**190.36**	102.55	Meropenem	613.8	509.41	639.28	601.58	602.72
Metronidazole	63.09	60.33	47.58	52.32	36.00	Metronidazole	359.01	316.41	293.89	259.75	231.69
Oxacillin	**145.52**	114.92	117.1	178.03	**149.14**	Oxacillin	**718.02**	557.74	579.35	627.01	**882.98**
Sulfamethoxazole/Trimethoprim	74.42	77.82	100.6	176.83	107.19	Sulfamethoxazole/Trimethoprim	475.2	515.55	619.71	**780.73**	772.85
Vancomycin	**176.02**	**166.40**	**181.72**	**241.48**	**139.66**	Vancomycin	**814.88**	**773.91**	**820.56**	**809.67**	878.52

DDD: defined daily dose; the top three antimicrobials per year are in bold.

**Table 3:** Antimicrobial consumption (J01, J02, J05) in tertiary care hospitals in Costa Rica from 2017 to 2021, expressed in DDD/100 bed days and DDD/100 discharges.

Total	2017	2018	2019	2020	2021	% AAPC 2017–2021	AAPC 95% CI	Trend (β1)	*p* Value
**Total antimicrobials (J01, J02, J05)**									
**DDD/100 bed days**	61.35	54.30	60.13	93.05	61.78	0.14	(−32.11 to 32.39)	3.96	0.31
**DDD/100 discharges**	270.63	245.68	277.80	295.44	313.25	2.97	(−5.25 to 11.19)	13.50	**0.0007**
**Total antibiotics (J01)**									
**DDD/100 bed days**	30.36	27.92	28.67	40.86	25.51	−3.42	(−32.78 to 25.94)	0.32	0.84
**DDD/100 discharges**	164.29	149.18	155.53	154.64	161.92	0.29	(−5.85 to 5.27)	0.072	0.96
**Total antifungals (J02)**									
**DDD/100 bed days**	26.30	22.68	27.60	43.21	32.49	4.31	(−28.22 to 36.84)	3.29	0.05
**DDD/100 discharges**	80.41	73.59	94.49	104.61	123.52	8.97	(−4.67 to 22.61)	11.72	**<0.0001**
**Total antivirals (J05)**									
**DDD/100 bed days**	4.69	3.70	3.87	8.98	3.77	−4.27	(−74.42 to 65.88)	−0.34	0.57
**DDD/100 discharges**	25.93	22.91	27.78	36.19	27.81	1.41	(−21.12 to 23.94)	1.70	0.14

DDD: defined daily dose; %AAPC: average annual percent change; 95% CI: 95% confidence intervals; Trend β1: the estimated change in antimicrobial consumption per year; statistically significant *p* values are in bold.

**Table 4:** Antimicrobial consumption (J01, J02 and J05) trends for the main groups of antimicrobials from 2017 to 2021, expressed in DDD/100 bed days and DDD/100 discharges.

Total	2017	2018	2019	2020	2021	%AAPC 2017–2021	AAPC 95% CI	Trend (β1)	*p* Value
Tetracyclines (J01A)									
DDD/100 bed days	15.14	13.83	12.72	16.86	13.78	−0.8	−18.0 to 16.4	0.031	0.94
DDD/100 discharges	88.75	93.84	91.26	91.37	88.57	0.0	−3.1 to 3.1	−0.28	0.63
Penicillins (J01C)									
DDD/100 bed days	264.03	218.55	233.13	339.47	221.26	0.0	−26.4 to 26.4	3.53	0.80
DDD/100 discharges	1390.00	1165.89	1212.08	1278.22	1344.66	−0.4	−8.4 to 7.6	2.16	0.93
Cephalosporis (J01D)									
DDD/100 bed days	430.66	405.92	369.09	485.73	296.08	−5.8	−27.8 to 16.2	−18.93	0.29
DDD/100 discharges	2495.15	2313.39	2239.63	1999.53	1946.72	−6.0	−8.8 to −3.1	−141.07	**<0.0001**
Carbapenems, monobactams (J01DH, J01DF)									
DDD/100 bed days	146.13	147.72	172.54	260.09	156.77	6.6	−21.7 to 35.0	13.36	0.27
DDD/100 discharges	699.00	644.69	791.78	793.57	948.00	7.6	−2.7 to 17.9	64.68	**<0.0001**
Macrolides, lincosamides (J01F)									
DDD/100 bed days	50.42	61.99	44.17	46.65	25.39	−14.3	−40.0 to 11.4	−6.54	**0.005**
DDD/100 discharges	360.60	347.88	305.84	240.73	174.26	−16.5	−24.4 to −8.7	−47.98	**<0.0001**
Sulfonamides and trimethoprim (J01E)									
DDD/100 bed days	74.42	77.82	100.60	176.83	107.19	15.9	−20.3 to 52.1	16.45	0.06
DDD/100 discharges	475.60	515.66	616.71	780.73	772.85	12.6	3.7 to 21.5	85.95	**<0.0001**
Aminoglycosides (J01G)									
DDD/100 bed days	69.62	56.59	63.76	88.93	71.01	3.0	−18.5 to 24.5	3.51	0.24
DDD/100 discharges	339.28	258.60	307.13	277.29	419.03	8.4	−16.6 to 33.3	17.81	0.26
Quinolones (J01M)									
DDD/100 bed days	68.01	56.02	59.38	113.04	84.09	13.2	−27.1 to 53.5	8.91	0.08
DDD/100 discharges	389.65	332.23	329.36	424.43	527.55	9.4	−6.3 to 25.1	36.80	**0.02**
Other antibacterials (J01XA01): Vancomycin									
DDD/100 bed days	176.02	166.40	181.72	241.48	139.66	−1.6	−25.4 to 22.2	0.23	0.98
DDD/100 discharges	814.88	773.91	820.56	809.67	878.52	2.0	−2.8 to 6.7	16.30	**0.03**
Other antibacterials (J01XX08): Linezolid									
DDD/100 bed days	46.41	28.18	47.76	76.56	34.61	1.8	−41.7 to 45.3	2.47	0.62
DDD/100 discharges	175.82	121.69	185.60	190.10	202.50	3.1	−17.1 to 23.4	12.17	0.08
Azole Antifungals (J02AB, J02AC)									
DDD/100 bed days	116.52	105.78	124.21	179.35	163.64	10.3	−8.9 to 29.6	16.78	**0.0008**
DDD/100 discharges	647.18	599.22	778.78	822.70	1045.72	12.1	0.0 to 24.2	102.05	**<0.0001**
Echinocandins Antifungals (J02AX)									
DDD/100 bed days	41.26	30.28	41.36	79.92	31.33	8.2	−42.9 to 59.2	2.97	0.59
DDD/100 discharges	156.94	136.72	166.16	223.39	189.48	6.0	−12.3 to 24.4	15.17	**0.01**
Antivirals (J05A)									
DDD/100 bed days	23.45	18.49	19.34	44.88	18.86	14.3	−48.3 to 77.0	1.72	0.57
DDD/100 discharges	25.93	22.91	27.78	38.19	27.81	4.0	−18.0 to 26.0	1.70	0.14

DDD: defined daily dose (unadjusted); % AAPC: average annual percent change, 95% CI: 95% confidence intervals; Trend β1: the estimated change in antimicrobial consumption per year; statistically significant *p* values are in bold.

**Table 5:** Antibiotic consumption trends according to WHO AWaRe classification, expressed in DDD/100 bed days and DDD/100 discharges.

Total	2017	2018	2019	2020	2021	% AAPC 2017–2021	AAPC 95% CI	Trend (β1)	*p* Value
**ACCESS**									
**DDD/100 bed days**	621.18	553.66	539.78	748.08	519.41	−4.4	−47.6 to 45.0	−0.91	0.97
**DDD/100 discharges**	3452.78	3173.04	3161.55	3030.79	3209.69	−1.8	−22.2 to 7.8	−62.84	0.05
**WATCH**									
**DDD/100 bed days**	746.69	723.13	752.68	1079.66	623.95	−4.4	−55.3 to 56.4	11.1	0.81
**DDD/100 discharges**	3872.60	3550.61	3809.19	3992.44	3981.68	0.7	−9.6 to 11.3	55.99	0.14
**RESERVE**									
**DDD/100 bed days**	58.42	35.33	54.68	92.34	55.34	−1.3	−82.8 to 104.8	5.08	0.34
**DDD/100 discharges**	230.83	152.55	225.53	249.4	328.01	9.2	−42.3 to 70.3	29.12	**0.016**

WHO AWaRe: World Health Organization antibiotic classification (access, watch and reserve); DDD: defined daily dose; %AAPC: average annual percent change; 95% CI: 95% confidence intervals; Trend β1: the estimated change in antimicrobial consumption per year; statistically significant *p* values are in bold.

## Data Availability

The data presented in this study are available on request from the corresponding author.
